# Variation in the immune responses against *Plasmodium falciparum* merozoite surface protein-1 and apical membrane antigen-1 in children residing in the different epidemiological strata of malaria in Cameroon

**DOI:** 10.1186/s12936-017-2105-4

**Published:** 2017-11-09

**Authors:** Tebit Emmanuel Kwenti, Adzemye Linus Moye, Adzemye Basil Wiylanyuy, Longdoh Anna Njunda, Theresa Nkuo-Akenji

**Affiliations:** 1 Department of Microbiology and Parasitology, University of Buea, P.B. 63, Buea, Cameroon; 2 Department of Medical Laboratory Sciences, University of Buea, P.B. 63, Buea, Cameroon; 3 District Hospital of Cite Verte, P.B. 3604, Yaounde, Cameroon; 4 District Hospital of Mbengwi, Mbengwi, North West Region Cameroon

**Keywords:** *Plasmodium falciparum*, Merozoite surface protein-1, Apical membrane antigen-1, Malaria immune responses, ELISA, Children, Epidemiological strata, Cameroon

## Abstract

**Background:**

Studies to assess the immune responses against malaria in Cameroonian children are limited. The purpose of this study was to assess the immune responses against *Plasmodium falciparum* merozoite surface protein-1 (MSP-1_19_) and apical membrane antigen-1 (AMA-1) in children residing in the different epidemiological strata of malaria in Cameroon.

**Methods:**

In a cross-sectional survey performed between April and July 2015, 602 children between 2 and 15 years (mean ± SD = 5.7 ± 3.7), comprising 319 (53%) males were enrolled from five epidemiological strata of malaria in Cameroon including: the sudano-sahelian (SS) strata, the high inland plateau (HIP) strata, the south Cameroonian equatorial forest (SCEF) strata, the high western plateau (HWP) strata, and the coastal (C) strata. The children were screened for clinical malaria (defined by malaria parasitaemia ≥ 5000 parasites/µl plus axillary temperature ≥ 37.5 °C). Their antibody responses were measured against *P. falciparum* MSP-1_19_ and AMA-1 vaccine candidate antigens using standard ELISA technique.

**Results:**

A majority of the participants were IgG responders 72.1% (95% CI 68.3–75.6). The proportion of responders was higher in females (p = 0.002) and in children aged 10 years and above (p = 0.005). The proportion of responders was highest in Limbe (C strata) and lowest in Ngaoundere (HIP strata) (p < 0.0001). Similarly, the mean IgG antibody levels were higher in children aged 10 years and above (p < 0.0001) and in Limbe (p = 0.001). The IgG antibody levels against AMA-1 were higher in females (p = 0.028), meanwhile no gender disparity was observed with MSP-1. Furthermore the risk of clinical malaria (p < 0.0001) and the mean parasite density (p = 0.035) were higher in IgG non-responders.

**Conclusion:**

A high proportion of IgG responders was observed in this study, suggesting a high degree exposure of the target population to malaria parasites. The immune responses varied considerably across the different strata: the highest levels observed in the C strata and the lowest in the HIP strata. Furthermore, malaria transmission in Cameroon could be categorized into two major groups based on the serological reaction of the children: the southern (comprising C and SCEF strata) and northern (comprising HWP, HIP and SS strata) groups. These findings may have significant implications in the design of future trials for evaluating malaria vaccine candidates in Cameroon.

## Background

Malaria constitutes a major health problem in Cameroon where it accounts for 48% of all hospital admission, 30% of morbidity and 67% of childhood mortality per year [1, 2]. Malaria in Cameroon is caused predominantly by *Plasmodium falciparum* but other species, including *Plasmodium vivax*, *Plasmodium malariae* and *Plasmodium ovale* have also been reported to cause malaria albeit at lower frequencies [3–6]. The entire Cameroon’s population of over 22 million is at risk of infection [7]. The epidemiology of malaria in Cameroon is complex and has been described as “Africa in miniature” [8] because she has all the epidemiological strata of malaria present in Africa. Six epidemiological strata of malaria have been identified and mapped in Cameroon: the sudano-sahelian (SS) strata, high inland plateau (HIP) strata, savannah-forest transmission (SFT) strata, south Cameroonian equatorial forest (SCEF) strata, high western plateau altitude (HWP) strata, and the coastal (C) strata [9]. These epidemiological strata differ in terms of their geographical and ecological characteristics, transmission pattern and endemicity level, and in terms of the main vectors transmitting malaria parasites [9].

Children in Cameroon, as in most other endemic countries in Africa, are most at risk of clinical and severe malaria because of their low immunity [10–12]. Immunity to malaria is known to be acquired slowly and in an age-dependent manner, after repeated exposure [10]. At a later stage, the capability of controlling parasitaemia in the blood is developed. The mechanism underlying development of anti-disease immunity and factors governing effective protection are still largely unknown as findings from different correlates of antibody-mediated immunity studies are often conflicting in their conclusions [13, 14]. Presently there is no single immunological correlate of protection to clinical malaria, moreover those described do not sufficiently account for the overall variation in susceptibility observed in a population [14].

Several blood-stage antigens of the malaria parasite with different structure and location have been evaluated for their role in inducing protective antibodies against clinical malaria, including the merozoite surface proteins (MSP-1, MSP-2, MSP-3, etc.), the apical membrane antigen-1 (AMA-1), erythrocytes binding antigen (EBA-175 RII) and the glutamate-rich protein (GLURP) [15–20]. In this study, the immune responses against MSP-1 and AMA-1 was evaluated because they have been shown to have a strong association with protection against clinical malaria [21, 22] and there is a need to collect more information on natural immune responses in children living in a high malaria endemic area, such as Cameroon. The MSP-1 is one of the best characterized proteins in several *Plasmodium* spp. MSP-1 is the most abundant merozoite surface protein, and is thought to be involved in the initial attachment of the merozoite to the erythrocyte surface [22]. The 19 kDa C-terminal fragment of MSP-1 (MSP-1_19_) has been recognized as the target of immunoglobulin G (IgG)-based protective immunity [23] and is a promising vaccine candidate [24]. MSP-1_19_ was selected for this study over the other MSP-1 molecules because of the fine specificity of MSP-1_19_ specific antibodies [25, 26] coupled to its role in protecting against clinical malaria. The recombinant 62 kDa apical membrane antigen-1 (AMA-1), on the other hand, is present in both the pre-erythrocytic and asexual blood-stage forms of the *Plasmodium* parasite. AMA-1 is involved in the re-orientation of merozoites prior to invasion of the erythrocyte and thereby plays a central role in erythrocyte invasion by *Plasmodium* species [22, 27]. Antibodies against this molecule display inhibitory activities against sporozoite invasion of hepatocytes [28] and against merozoite invasion of erythrocytes [29, 30]. Sero-epidemiological studies performed in other malaria-endemic areas have shown some evidence of antibodies against these antigens in protecting against malaria [20]. However, studies designed to assess the immune responses to malaria parasites in children in Cameroon are limited, with a few studies published thus far describing only the immune responses in neonates and infants [31, 32].

Previous studies in Cameroon, have shown that malaria transmission decreases steadily northward, from the C strata in the south to the SS strata in the north; meanwhile the rate of severe malaria attack and mortality rate appears to increase in the same direction [33, 34]. It was hypothesized that the observed trend could be attributed to the decreasing immunity against *P. falciparum* as a result of the decrease in transmission intensity as one move towards the north. In order to test this hypothesis, the humoral immune responses (total IgG) against the *P. falciparum* MSP-1 and AMA-1 antigens was measured and compared in children in five epidemiological strata of malaria in Cameroon. This will generate baseline data that may be useful for designing studies for future testing of malaria vaccine candidates.

## Methods

### Study area

**Fig. 1:**
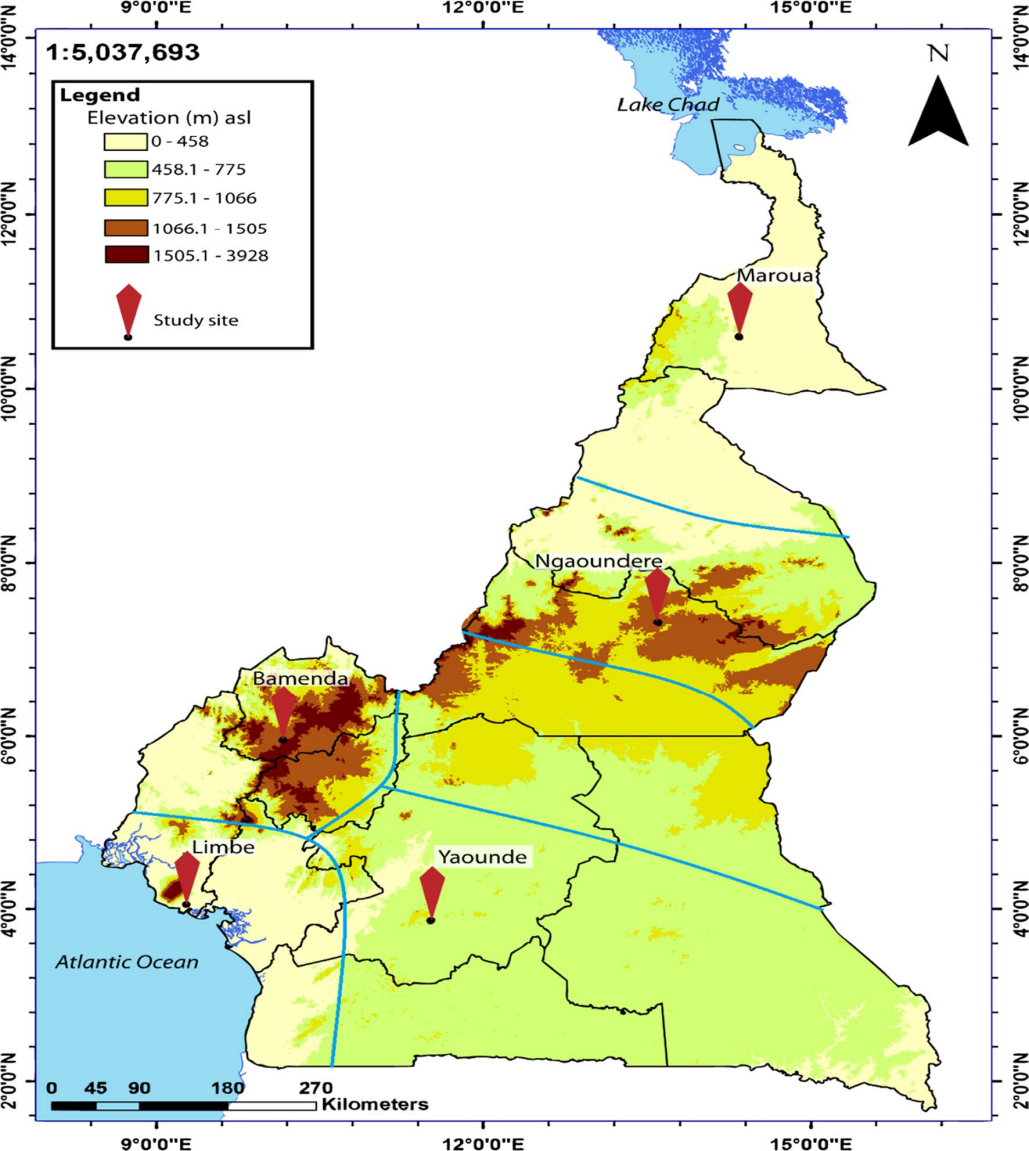
Map depicting the study sites selected. Five epidemiological strata are delineated

For this study, 5 out of the 6 epidemiological strata of malaria in Cameroon were randomly selected. Five study sites, each representing the epidemiological strata were further selected and included: Maroua in the SS strata, Ngaoundere in the HIP strata, Yaounde in the SCEF strata, Bamenda in the HWP strata, and Limbe in C strata (Fig. 1).

Bamenda (N = 5°56′ E = 10°10′) has an average elevation of 1614 m, with the Equatorial climate of the Cameroon type characterized by a fairly constant temperature ranging from 20.1 °C in July to 23.0 °C in March (mean = 21.5 °C), annual rainfall of 2145 mm, ranging from 9 mm in January to 383 mm in September, and two seasons: one dry (October–March) and one rainy (March–September). Malaria transmission in Bamenda can be described as meso-endemic (Kwenti et al. [33, 34]), with maximal transmission occurring at the beginning and towards the end of the rainy season. *Anopheles gambiae* is the principal malaria vector [9].

Limbe (N = 4°01′ E = 9°13′) has an average elevation of 150 m. The climate is dominated by the Equatorial climate of high rainfall and high temperature. Average annual temperature of Limbe is 26.5 °C, ranging from 15.8 °C in July to 32.8 °C in February. Limbe receives 1174 mm of rainfall annually, ranging from 27 mm of rain in January to 617 mm in August; relative humidity is constantly high (75–80%). Limbe has two seasons: one dry (October–February) and one rainy (March–October). Malaria transmission in Limbe can be described as hyperendemic [33, 34], with transmission being highest at the beginning and towards the end of the rainy season. The principal vector of malaria in Limbe is *An. gambiae* [9]. The entomological inoculation rate in this area has been estimated at 161 bites per person per year [35].

Yaoundé (3°52′N 11°31′E) has an average elevation of 750 m, with the Guinea-type Equatorial climate [36], characterized by fairly constant temperatures ranging from 22.6 °C in August to 24.6 °C in March (mean = 23.7 °C), average annual rainfall of 1643 mm, average relative humidity index ranging from 85 to 90%, and four distinct seasons: two rainy (March–May/June, September–November) and two dry (December–February, June/July–August). Maximal transmission of malaria occurs during and immediately following the two rainy seasons [36, 37]. Malaria transmission in Yaoundé is holo-endemic [33, 34] and seasonal, with *An. gambiae* and *Anopheles funestus* as the main vectors. The entomologic inoculation rate has been estimated at 34 infectious bites per person per year [38].

Ngaoundere (N = 7°19′00″ E = 13°35′00″) with an average elevation of 1212 m, has the Sudani-Guinean tropical climate, characterized by fairly constant temperature averaging 22.1 °C, average annual rainfall of 1485 mm, ranging from 0 mm in December to 280 mm in August. Ngaoundere has two distinct seasons: one dry (October–April) and one rainy (May–September). Malaria transmission can be described as meso-endemic [33, 34] with seasonal outbreaks common. *Anopheles gambiae* is the principal malaria vector [9].

Maroua (N = 10°35′50″ E = 14°18′57″) has an average elevation of 384 m. The climate is considered to be sudano-sahelian. There is not much of rainfall all year long. The average annual rainfall is 794 mm, ranging from 0 mm in January to 245 mm in August. The average temperature is 35 °C [39]. April is the warmest month of the year (45 °C). Maroua has two distinct seasons: a short rainy season (July–October) and a long dry season (November–June). Malaria transmission in Maroua can be described as hypo-endemic [33, 34] and unstable with a risk of epidemic. The principal malaria vector is *An. gambiae*; *Anopheles arabiensis* along with *An. funestus* have also been identified here [9]. The entomologic inoculation rate has been estimated at 18.25 infectious bites per person per year [39].

### Study design and duration

This was a cross-sectional survey performed in the 5 epidemiological strata described above. Data were collected simultaneously in the different study sites between 5 April and 7 July, 2015 (to coincide with the rainy season during which transmission is highest).

### Sample size estimation

Using the effect size of 0.18 calculated from data obtained by Nebie et al. [40], the power of study 0.8 considering alpha 0.05, and the ANOVA function in G*Power, obtained a sample size of 75 participants per study site giving a total of at least 375 (5 × 75).

### Study population

Children residing in the different study sites were randomly selected to take part in the study. The participants were between 2 and 15 years of age and of both gender. The participants had been residing in the study site for at least 2 years. Children who did not meet these criteria were excluded from the study.

### Laboratory analysis

#### Specimen collection

About 4 ml of blood was collected from consented participants using aseptic techniques into EDTA and dry tubes. Blood in the EDTA tubes were used for the performance of the complete blood count (CBC) and preparation of thick and thin blood films for malaria screening. Aliquots of the serum from the dry test tubes were transferred into Eppendorf tubes and stored at − 40 °C for the performance of ELISA in the future.

#### Performance of complete blood count

CBC was performed using the Mindray^®^ Auto haematology analyzer (BC-2800, Shenzhen Mindray Bio-Medical Electronics Co. Ltd). The white blood cell counts were obtained from the CBC results and used in the estimation of the malaria parasite density.

#### Detection of malaria parasites

Prepared blood films were air-dried and stained with 10% Giemsa (1 in 20 dilution) for 25–30 min [41] and examined by light microscopy. Detection of malaria parasite and estimation of the parasite density was performed in accordance with the proceedings of the Research Malaria Microscopy Standards Working Group [42]. Blood films were read by two expert microscopists who were blinded from the results of the other. In case of any discrepancy obtained by the two microscopists, a third was brought in and the results obtained was considered as final. At least 200 fields were screened using the 100× (oil immersion) objective. If asexual stages of *Plasmodium* spp. were seen, they were counted until 500 white blood cells (WBC) were reached. The slides were only declared negative after counting to 2500 WBC. Estimation of the parasite density was done by dividing the parasites counted by 500 and then multiplied by the WBC count of the participants to give numbers in parasite per μl [42].

#### Measurement of humoral immune response in the study population

The IgG responses against MSP-1_19_ (19-kDa C-terminal fragment, 3D7 strain) and AMA-1 (ectodomain, 3D7 strain), kindly provided by Andreas Latz (NovaTec, Immundiagnostica, GmbH), were assessed by ELISA technique following a standardized methodology described in the Afro-immunoassay network standard operating procedure (procedure number AIA-001-02) [19] with modification; pooled positive sera, captured by coated antigens, were used as standard calibrators in place of capture monoclonal antibodies used in the original AIA protocol. Briefly, to microtitre plates coated with recombinant protein (MSP-1 and AMA-1) was added sera samples (diluted 1 in 100 and 1 in 1000 for MSP-1 and AMA-1, respectively) along with positive control serum (a pool of sera from 8 adults in Muyuka with lifelong exposure to malaria) and 10 negative control sera from non-exposed German adults. At the end of the reaction, absorbance was read at 450 nm with an ELISA plate reader, BioTek^®^ ELx800TM (BioTek Instruments, Inc., USA). Antibody responses were converted to arbitrary units (AU) with the aid of a standard curve derived from serial dilution (1:200, 1:400, 1:800, 1:1600, and 1: 3200) of positive control sera for all test plates, with the absorbance of the lowest dilution corresponding to 100 AU. The cut-off for positivity was defined as the AU value 3 standard deviations above the arithmetic mean (which was 11.907 ± 3.55) for the negative control sera.

### Statistical analysis

Data collected were entered into an Excel spreadsheet and analysed using the Stata^®^ version 12.1 software (StataCorp LP, Texas, USA) and IBM^®^ SPSS^®^ Statistics version 20. Data were log-transformed prior to statistical analysis. The statistical tests performed included the Pearson Chi square for categorical variables, Student’s *t* test and ANOVA for the comparison of group means, and multivariate regression analysis for the determination of associations between groups adjusting for possible confounding. Statistical significance was set at p < 0.05. Clinical malaria was defined as malaria parasitaemia (≥ 5000 parasites/µl) plus fever (axillary temperature ≥ 37.5 °C).

## Results

### Characteristics of study population

Six-hundred and two (602) children consented and participated in the study. Among them were 319 (53.0%) males and 283 (47.0%) females (Table 1). The mean (± SD) age of the participants was 5.7 (± 3.7) years.

**Table 1:** Distribution of the study population by age, gender and study site

Study site	Age category (years)			Total
	< 5	5–9	≥ 10	
Bamenda				
Gender				
F	27 (43.5)	16 (25.8)	19 (30.6)	62 (46.6
M	31 (43.7)	26 (36.6)	14 (19.7)	71 (53.4)
Total	58 (43.6)	42 (31.6)	33 (24.8)	133
Limbe				
Gender				
F	32 (42.7)	29 (38.7)	14 (18.7)	75 (53.6)
M	38 (58.5)	19 (29.2)	8 (12.3)	65 (46.4)
Total	70 (50.0)	48 (34.3)	22 (15.7)	140
Maroua				
Gender				
F	21 (63.6)	12 (36.4)	0 (0.0)	33 (43.4)
M	25 (58.1)	15 (34.9)	3 (7.0)	43 (56.6)
Total	46 (60.5)	27 (35.5)	3 (4.0)	76
Ngaoundere				
Gender				
F	39 (63.9)	17 (27.9)	5 (8.2)	61 (48.4)
M	39 (60.0)	18 (27.7)	8 (12.3)	65 (51.6)
Total	78 (61.9)	35 (27.8)	13 (10.3)	126
Yaounde				
Gender				
F	13 (25.0)	25 (48.1)	14 (26.9)	52 (40.9)
M	27 (36.0)	27 (36.0)	21 (28.0)	75 (59.1)
Total	40 (31.5)	52 (40.9)	35 (27.6)	127
Total				
Gender				
F	132 (46.6)	99 (35.0)	52 (18.4)	283 (47.0)
M	160 (50.2)	105 (32.9)	54 (16.9)	319 (53.0)
Total	292 (48.5)	204 (33.9)	106 (17.6)	602

Overall, clinical malaria was observed in 88 of the 602 participants giving a prevalence of 14.6% (95% CI 11.9–17.7). Malaria prevalence was highest in Limbe (22.1%) and lowest in Maroua (5.6%) (Table 2). A significant association was observed between prevalence of clinical malaria and study site (p < 0.0001) adjusting for age and gender. All (100%) cases of malaria were caused by *P. falciparum*. The overall geometric mean parasite density (GMPD) observed in this study was 24,690.5 parasites/μl. The GMPD (in parasites/μl) were 11,620.8, 24,952.9, 189,453.8, 489.3, and 7262.9 for Bamenda, Limbe, Yaounde, Maroua, and Ngaoundere, respectively. A significant association was observed between GMPD and study site (p < 0.0001).

**Table 2:** Distribution of clinical malaria in the study population stratified according to study site, age and gender

Parameter	n	Clinical malaria present n (%)	Univariate analysis		Multivariate analysis	
			χ^2^	p value	^a^χ^2^	p value
Study site						
Bamenda	133	18 (13.5)	13.745	0.008		
Limbe	140	31 (22.1)				
Yaounde	127	22 (17.3)				
Maroua	72	4 (5.6)			73.866	< 0.0001
Ngaoundere	126	13 (10.3)				
Total	602	88 (14.6)				
Age (years)						
< 5	292	32 (11.0)	7.221	0.027		
5–9	204	40 (19.6)				
≥ 10	106	16 (15.1)			7.521	0.023
Total	602	88 (14.6)				
Gender						
Females	283	48 (16.9)	2.349	0.125		
Males	319	40 (12.5)			0.254	0.614
Total	602	88 (14.6)				

^a^Chi square values obtained from the likelihood ratio tests

There was no significant difference in the prevalence of clinical malaria between males and females (p = 0.614) adjusting for age and study site (Table 2).

The prevalence of clinical malaria was higher in children between 5 and 9 years (19.6%) and lowest in children below 5 years (11.0%) (Table 2). A significant association was observed between prevalence of clinical malaria and age (p = 0.023) adjusting for study site and gender.

### Distribution of antibody seropositivity in the study population

A majority of participants were IgG responders 434 (72.1%, 95% CI 68.3–75.6). The proportion of IgG responders varied between study sites, being highest in Limbe (91.4%) and lowest in Ngaoundere (53.2%) (Table 3). The variation in the proportion of IgG responders across the different study sites was similar between the two recombinant antigens used. Overall, the association between the proportion of responders and study sites was observed to be statistically significant (p < 0.0001) adjusting for age and gender.

**Table 3:** The distribution of the proportion of IgG responders stratified according to study site

Recombinant antigen	Responders	Study site					Total n (%)	Univariate analysis		Multivariate analysis	
		Bamenda n (%)	Limbe n (%)	Yaounde n (%)	Maroua n (%)	Ngaoundere n (%)		χ²	p value	χ²	p value
AMA-1	Positive	53 (39.9)	79 (56.4)	68 (53.5)	27 (35.5)	23 (18.3)	242 (40.2)	49.741	< 0.0001		
	Negative	80 (60.1)	61 (43.6)	59 (46.5)	49 (64.5)	103 (81.7)	360 (59.8)				
	Total	133	140	127	76	126	602				
MSP-1	Positive	72 (54.1)	104 (74.3)	91 (71.7)	36 (47.4)	49 (38.9)	352 (58.5)	48.294	< 0.0001		
	Negative	61 (45.9)	36 (25.7)	36 (28.3)	40 (52.6)	77 (61.1)	250 (41.5)				
	Total	133	140	127	76	126	602				
Overall	Positive	89 (66.9)	128 (91.4)	107 (84.3)	43 (56.6)	67 (53.2)	434 (72.1)	68.626	< 0.0001	67.840^a^	< 0.0001
	Negative	44 (33.1)	12 (8.6)	20 (15.7)	33 (43.4)	59 (46.8)	168 (27.9)				
	Total	133	140	127	76	126	602				

^a^Chi square values obtained from the likelihood ratio tests adjusted for age and gender

Similarly the proportion of IgG responders varied with age; with the highest proportion of responders observed in children ≥ 10 years (82.1%) and the lowest proportion observed in children < 5 years (66.4%), irrespective of the recombinant antigen used (Table 4). Overall, the association between responders and age was observed to be significant (p = 0.009) adjusting for gender and study site.

**Table 4:** The distribution of the proportion of responders stratified according to age

Recombinant antigen	Responders	Age category (years)			Total n (%)	Univariate analysis		Multivariate analysis	
		< 5 n (%)	5–9 n (%)	≥ 10 n (%)		χ^2^	p value	χ^2^	p value
AMA-1	Positive	85 (29.1)	94 (46.1)	63 (59.4)	242 (50.5)	34.185	< 0.0001		
	Negative	207 (70.9)	110 (53.9)	43 (40.6)	360 (49.5)				
	Total	292	204	106	602				
MSP-1	Positive	160 (54.8)	117 (57.4)	75 (70.8)	352 (58.5)	8.317	0.016		
	Negative	132 (45.2)	87 (42.6)	31 (29.2)	250 (41.5)				
	Total	292	204	106	602				
Overall	Positive	194 (66.4)	153 (75.0)	87 (82.1)	434 (72.1)	10.748	0.005	7.567^a^	0.009
	Negative	98 (33.6)	51 (25.0)	19 (17.9)	168 (27.9)				
	Total	292	204	106	602				

^a^Chi square values obtained from the likelihood ratio tests adjusted for gender and study site

The proportion of IgG responders was observed to be higher in females 221/283 (78.1%) compared to males 213/319 (66.8%) irrespective of the recombinant antigen used. Multivariate analysis revealed a significant association between the proportion of responders and gender (p = 0.002) adjusting for age and study site.

### Comparison of total IgG antibody levels

**Fig. 2:**
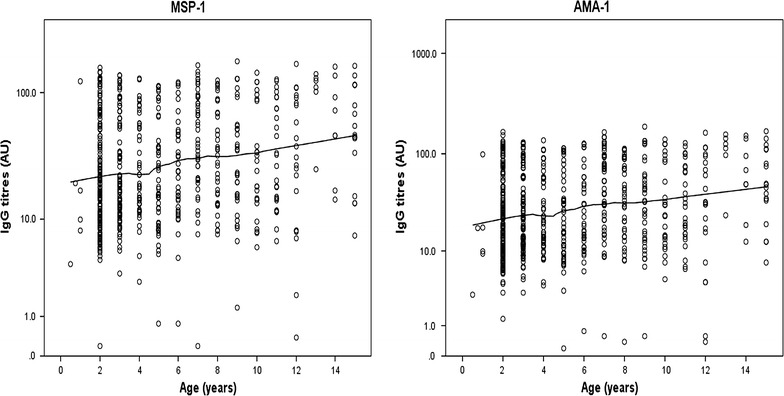
Variation of antibody level by age in the study population. The line shows the LOESS smoothed estimate of the geometric mean. Evidence show that antibody level increases with increasing age adjusting for gender and study site (p < 0.0001)

**Fig. 3:**
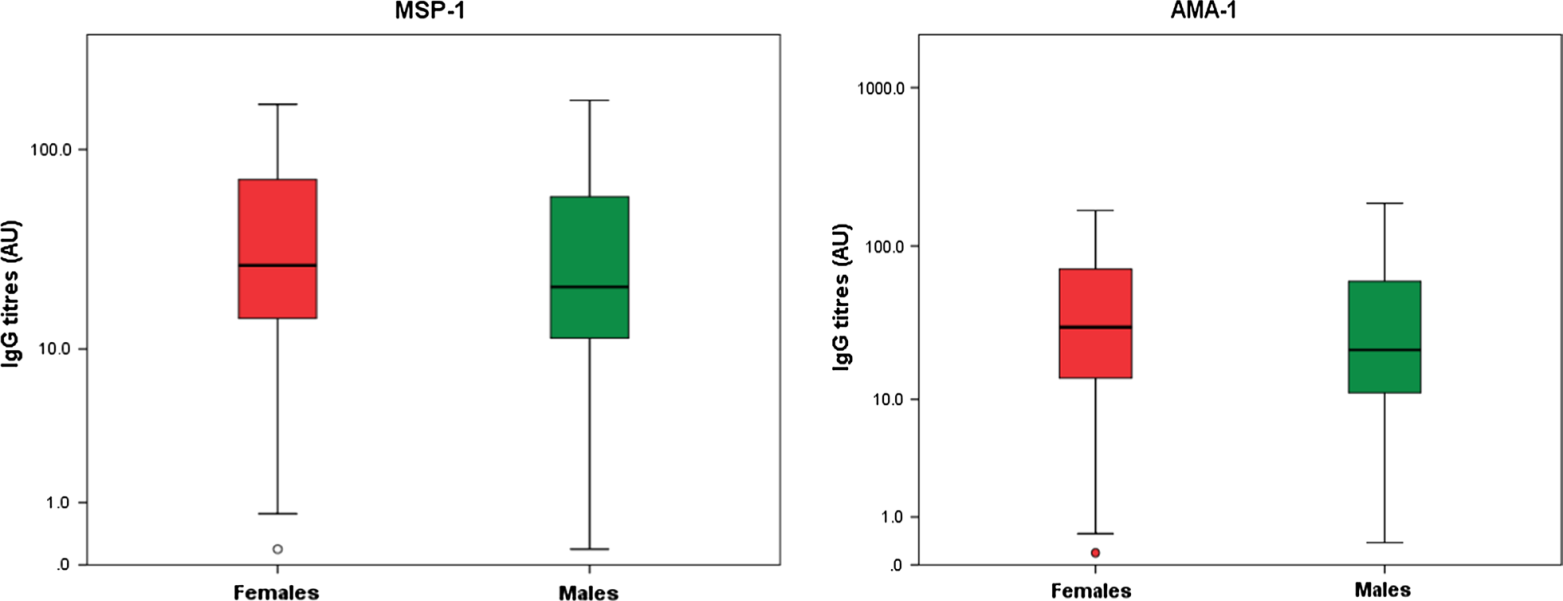
Box plot depicting the variation of mean antibody level (log-transformed) with gender. Females had a higher antibody level compared to males for AMA-1 (p = 0.010) but not for MSP-1 (p = 0.055) adjusting for age and study site

**Fig. 4:**
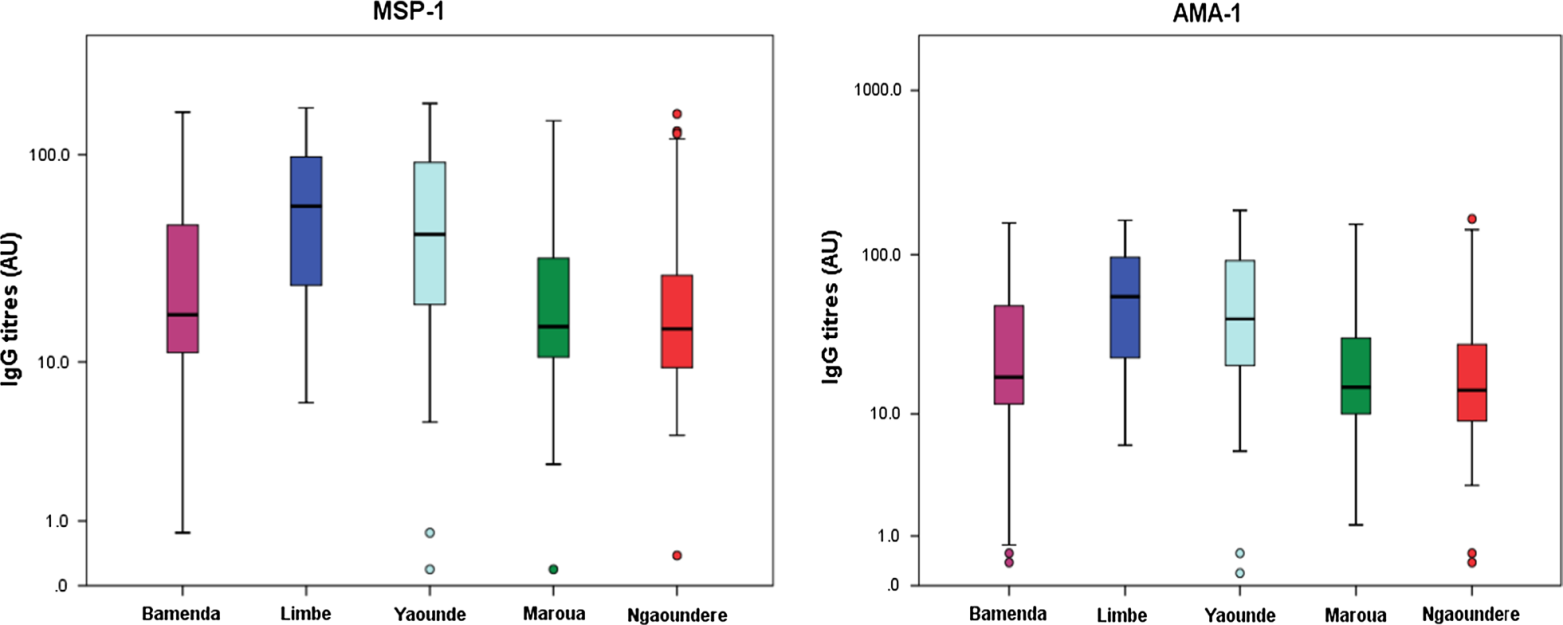
Box plot depicting the variation of mean antibody level (log-transformed) with study site. There is evidence of significant association between antibody level and the study site (p = 0.001) adjusting for age and gender

Overall antibody levels against MSP-1 and AMA-1 were observed to increase with increasing age (p < 0.0001) adjusting for gender and study site (Fig. 2). Antibody levels against MSP-1 was observed to be independent of gender (p = 0.055), meanwhile antibody levels against AMA-1 were significantly higher in females (p = 0.028) adjusting for age and study site (Fig. 3). The antibody levels against MSP-1 and AMA-1 were observed to vary with study site, being highest in Limbe and lowest in Ngaoundere (Fig. 4). Multiple linear regression revealed this association to be significant adjusting for age and gender (p ≤ 0.0001 for both MSP-1 and AMA-1).

**Fig. 5:**
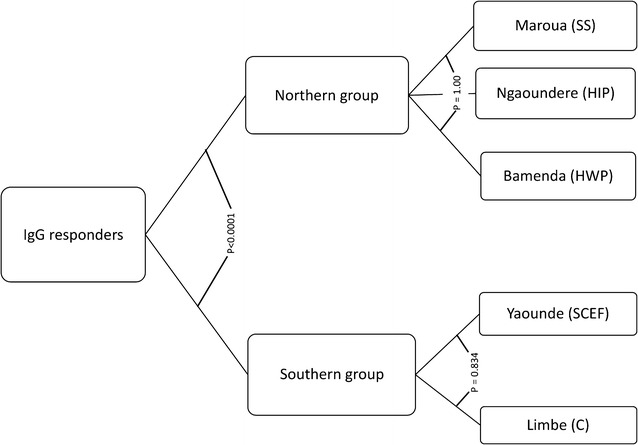
The serological reaction of children places the different epidemiological strata into two major groups: northern and southern. *C* coastal, *SCEF* South Cameroonian Equatorial forest, *HWP* high western plateau, *HIP* high inland plateau, *SS* sudano-sahelian

Significant differences were observed in the IgG antibody titres between Bamenda and Limbe (p < 0.0001); Bamenda and Yaoundé (p < 0.0001); Limbe and Maroua (p < 0.0001); Limbe and Ngaoundere (p < 0.0001); Yaounde and Maroua (p < 0.0001) and Yaounde and Ngaoundere (p < 0.0001). No significant differences were observed between Bamenda and Maroua (p = 1.00); Bamenda and Ngaoundere (p = 0.252); Maroua and Ngaoundere (p = 1.00) and between Limbe and Yaounde (p = 0.339). Based on the serological reaction of the children, the different epidemiological strata of malaria can be categorized further into two main groups: the southern (higher antibody titres) and the northern groups (lower antibody titres) (Fig. 5).

Prevalence of clinical malaria was observed to be higher among non-responders 46.1% (53/115) compared to responders 8.8% (35/399) (χ^2^ = 53.513, p < 0.0001). Furthermore, the risk of clinical malaria was observed to be higher in non-responders compared to responders (OR = 5.25, p < 0.0001).

**Fig. 6:**
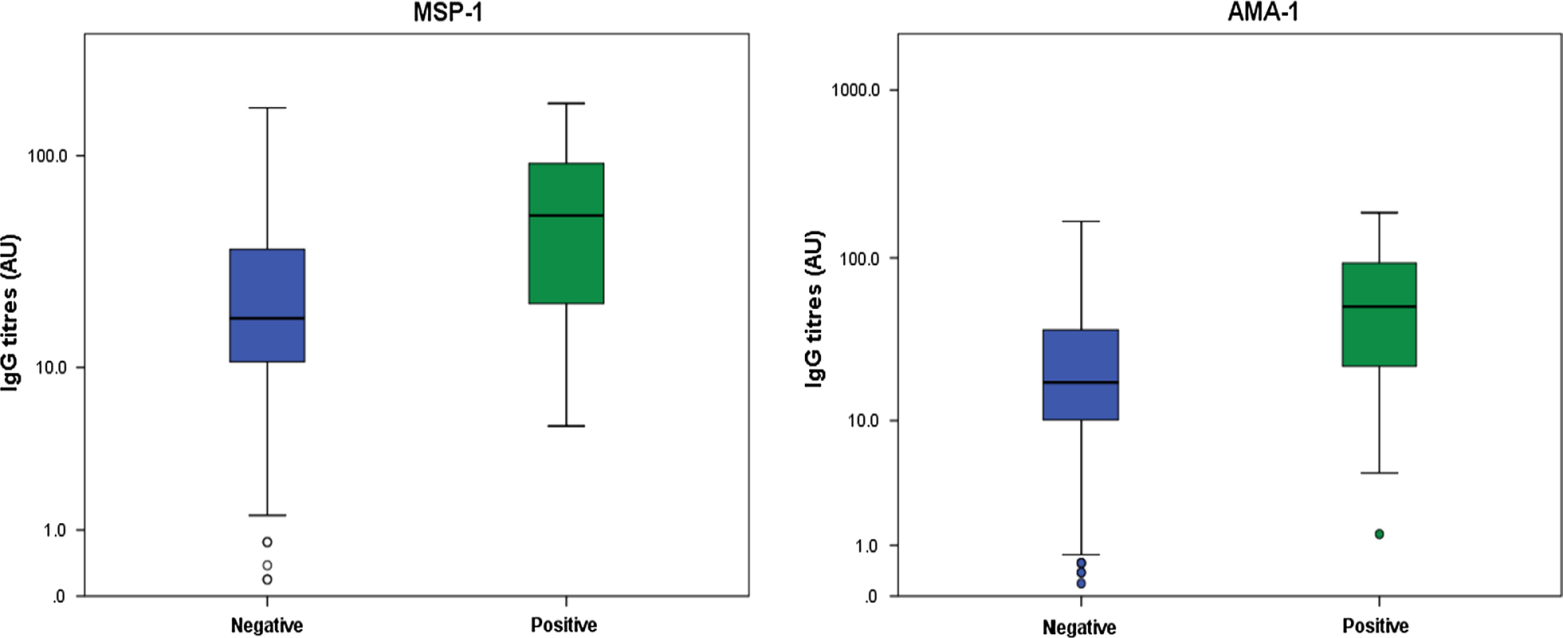
Box plot depicting the variation of antibody level (log-transformed) between infected and noninfected children. Evidence shows that antibody level was higher in infected children compared to non-infected children (p < 0.0001) adjusting for age, gender and study site

Overall, the mean antibody level against MSP-1 and AMA-1 was observed to be higher in children infected with malaria compared to children without (Fig. 6). The difference in the antibody level between infected and uninfected children was observed to be significant, adjusting for age, gender and study site (p < 0.0001) for both MSP-1 and AMA-1. Furthermore, the mean malaria parasite density was observed to be higher in non-responders (116,646.4 ± 180,426.7) compared to responders (54,208.87 ± 110,233.7), and this difference was significant (p = 0.035).

## Discussion

The proportion of IgG responders observed in this study was high (72.1%). These findings are similar to studies performed on different populations in Cameroon [3, 32]. The high proportion of IgG responders in this study could be due to the constant exposure to infective bites of mosquitoes or the participants sustained an immune response after active infection [3]. MSP-1 was recognized more often than AMA-1 (58.5 vs 40.2%) in the serum of the study participants. Studies have shown that the immunogenicity of the large repertoire of antigens often targeted as malaria vaccine candidates are not the same [13, 43]. However, these data should be interpreted with caution bearing in mind that the dilutions of the two antigens were not the same (1/100 for MSP-1 and 1/1000 for AMA-1). Furthermore, the proportion of responders was higher among females compared to males (p = 0.002). In addition, the mean IgG level was observed to be higher in females compared to males for AMA-1 (p = 0.010) but not with MSP-1 (p = 0.055). These findings suggest that in the study area females were more prone to infection with malaria parasites than males, which is evident from the finding of a higher malaria prevalence in females in this study, although it was not significant. The overall mean IgG antibody level in the current study was observed to increase with increasing age (p < 0.0001). These findings are in line with the numerous studies that show that immunity to malaria increases with age as the frequency of exposure also increases [10, 13, 40, 44].

In the current study, site-specific analysis revealed that the proportion of IgG responders was highest in Limbe (in C strata) and lowest in Ngaoundere (in the HIP strata) (p < 0.0001). Similarly the IgG antibody titres were observed to decrease northwards from Limbe (in C strata) where it peaked to Ngaoundere (in HIP strata) where it was lowest (p = 0.001). These observed differences could be attributed to the differences in the transmission intensity of malaria observed in the different epidemiological strata; malaria has been described as hyperendemic in Limbe [45], holo-endemic in Yaounde [46, 47], meso-endemic in Bamenda and Ngaoundere, and hypo-endemic in Maroua [33, 34]. The transmission of malaria in Cameroon has been observed to decrease steadily from the south towards the north of the country [33, 34]. The association between immunity to malaria and transmission intensity has previously been reported [35, 48–50]. The differences in immune responses could also be attributed to factors including ethnicity [45], red blood cell defects [51] or use of mosquito nets, which could greatly influence exposure to malaria parasites and modulate the levels of antibodies in different populations. The decreasing trend in the magnitude of antibody responses observed in the current study supports the hypothesis that increased risk of severe malaria and fatality towards the northern regions as earlier reported [33, 34] could be attributed to the decreasing immunity against malaria. Site-specific analysis revealed two major groups of malaria transmission based on the serological reaction of the children: the southern group (comprising C and SCEF strata) and the northern group (comprising the HWP, HIP and SS strata). The southern and northern groups differed remarkably in terms of their serological reaction (p < 0.0001), but the different strata that make up the two groups were similar to each other. These findings may have significant implications in the design of future trials to test malaria vaccine candidates in Cameroon. However, larger studies involving more study sites within the different epidemiological strata will be required to confirm these observations.

In the current study, the overall clinical malaria prevalence was 14.6%. This prevalence was lower than the national prevalence of 29% reported in 2012 [52] and could be attributed to the relentless effort of the Cameroon Government to control the disease through mass distribution of insecticide-treated bed nets to all households in the country and to the intense sensitization of the population through media [47, 53]. The prevalence of malaria was observed to decrease steadily northward from Limbe (C strata) where it peaked to Maroua (SS strata). A significant association was observed between prevalence of malaria and study site. This pattern in malaria prevalence could be related to differences in the level of transmission of malaria and explains the observed trend in the antibody responses. *Plasmodium falciparum* was identified as the only *Plasmodium* species causing malaria in the target population, which is in line with other studies [33, 34] but contradictory to studies that have reported other *Plasmodium* species causing malaria, including *P. vivax* [3, 4]. These discrepancies between this study and others could be attributed to differences in the study designs; this study targeted mainly children, meanwhile others targeted adults in addition to children.

The prevalence of malaria was significantly higher in non-responders compared to responders (p < 0.0001) in this study. Furthermore, non-responders were observed to be at a higher risk of clinical malaria compared to responders. These findings serves as confirmation of studies reporting that antibodies to MSP-1 and AMA-1 are associated with protection against clinical malaria [13, 31] and therefore reinforces their potential as promising vaccine candidates. In this study, the mean IgG antibody level was observed to be higher in children infected with malaria parasites compared to their non-infected counterparts (p < 0.0001). This suggests that anti-MSP-1 and anti-AMA-1 antibodies are highest at the presence of infection and decay shortly after treatment. This observation conforms to studies performed elsewhere [54–57]. Similarly, the mean parasite density was observed to be higher in non-responders compared to responders (p = 0.035). The observation of higher parasite density in non-responders conforms to the study by Taylor et al. [31] and suggests the role of antibodies in the protection against clinical malaria.

This study, which has revealed the pattern of the immune responses against malaria in Cameroonian children, is limited in that children were recruited in major urban centers only, and the findings may not be generalizable to children in rural areas. Only two malaria vaccine candidate antigens were used in this study, which may not be a true reflection of the overall immune responses in the target population. In addition, only total IgG antibody was measured and not the IgG sub-types. Larger studies involving many more malaria vaccine candidate antigens as well as measuring the different IgG sub-types will be required to give a clearer picture of the immunity against malaria parasites in Cameroonian children. Furthermore, the seasonal variation of the immune responses against malaria parasites was not investigated in the current study as participants were recruited during the rainy season only during which malaria transmission is usually higher. Transmission of malaria in Cameroon especially in the northern regions is seasonal and generally lower during the dry season. Studies designed to recruit children during the rainy and dry seasons will therefore be required to provide a clearer picture.

## Conclusion

Overall, a high proportion (72.1%) of children in the current study were IgG responders. IgG antibody responses against MSP-1 and AMA-1 recombinant antigens were observed to increase in magnitude with increasing age. The risk of clinical malaria in this study was observed to be higher in IgG non-responders compared to responders. Furthermore, malaria parasite density was significantly higher in non-responders compared to responders. The immune responses against MSP-1 and AMA-1 vaccine candidate antigens in the current study were observed to decrease steadily from Limbe (in C strata) to Ngaoundere (in HIP strata). Furthermore, based on the serological reaction of the children, malaria transmission in Cameroon could be categorized into two major groups: the southern (comprising C and SCEF strata) and the northern (comprising HWP, HIP and SS strata) groups. These findings may have significant implications in the design of future trials to test malaria vaccine candidates in the country.

## Abbreviations

SS: sudano-sahelian strata
HIP: high inland plateau strata
SCEF: South Cameroonian Equatorial strata
HWP: high western plateau strata
C: coastal strata
GMPD: geometric mean parasite density
MSP: merozoite surface protein
AMA: apical membrane antigen
Ig: immunoglobulin
